# Microarray-based comparative genomic hybridisation of breast cancer patients receiving neoadjuvant chemotherapy

**DOI:** 10.1038/sj.bjc.6603483

**Published:** 2006-11-28

**Authors:** J-Y Pierga, J S Reis-Filho, S J Cleator, T Dexter, A MacKay, P Simpson, K Fenwick, M Iravani, J Salter, M Hills, C Jones, A Ashworth, I E Smith, T Powles, M Dowsett

**Affiliations:** 1 Breakthrough Breast Cancer Research Center, The Institute of Cancer Research, London, SW3 6JB, UK; 2 Medical Oncology Department, Institut Curie, Paris, cedex 5, France; 3 Academic Department of Biochemistry, Royal Marsden Hospital NHS Trust, Fulham Road, London, SW3 6JJ, UK; 4 Section of Paediatric Oncology, Institute of Cancer Research, London, SM2 5NG, UK; 5 Breast Cancer Unit, Royal Marsden Hospital, London, SW3 6JJ, UK

**Keywords:** breast cancer, comparative genomic hybridisation, microarrays, neoadjuvant chemotherapy

## Abstract

We analysed the molecular genetic profiles of breast cancer samples before and after neoadjuvant chemotherapy with combination doxorubicin and cyclophosphamide (AC). DNA was obtained from microdissected frozen breast core biopsies from 44 patients before chemotherapy. Additional samples were obtained before the second course of chemotherapy (D21) and after the completion of the treatment (surgical specimens) in 17 and 21 patients, respectively. Microarray-based comparative genome hybridisation was performed using a platform containing ∼5800 bacterial artificial chromosome clones (genome-wide resolution: 0.9 Mb). Analysis of the 44 pretreatment biopsies revealed that losses of 4p, 4q, 5q, 12q13.11–12q13.12, 17p11.2 and 17q11.2; and gains of 1p, 2p, 7q, 9p, 11q, 19p and 19q were significantly associated with oestrogen receptor negativity. 16q21–q22.1 losses were associated with lobular and 8q24 gains with ductal types. Losses of 5q33.3–q4 and 18p11.31 and gains of 6p25.1–p25.2 and Xp11.4 were associated with *HER2* amplification. No correlations between DNA copy number changes and clinical response to AC were found. Microarray-based comparative genome hybridisation analysis of matched pretreatment and D21 biopsies failed to identify statistically significant differences, whereas a comparison between matched pretreatment and surgical samples revealed a statistically significant acquired copy number gain on 11p15.2–11p15.5. The modest chemotherapy-driven genomic changes, despite profound loss of cell numbers, suggest that there is little therapeutic selection of resistant non-modal cell lineages.

Breast cancer is a heterogeneous disease comprising tumours with remarkably distinct clinical behaviour (Simpson *et al*, 2005). In the last 20 years, a multitude of prognostic and predictive markers have been tested, however only oestrogen receptor, progesterone receptor and HER2 are currently used to tailor the therapy of breast cancer patients (Goldhirsch *et al*, 2005). The use of expression arrays to derive molecular profiles that are predictive of clinical outcome has received great attention in the last 5 years (van't Veer *et al*, 2005; Reis-Filho *et al*, 2006b), especially in the neoadjuvant setting (Davidson and Morrow, 2005). Predictive signatures for different neoadjuvant chemotherapy regimens have been reported by some investigators but with less success by others (Chang *et al*, 2003; Ayers *et al*, 2004; Cleator and Ashworth, 2004; Chang *et al*, 2005a; Hannemann *et al*, 2005; Iwao-Koizumi *et al*, 2005; Modlich *et al*, 2005; Rouzier *et al*, 2005; Dressman *et al*, 2006; Reis-Filho *et al*, 2006b). Although some of these results are promising, issues related to the instability of mRNA, experimental design and data analysis have led many to call into question the validity of current approaches (Ransohoff, 2004; Brenton *et al*, 2005; Ioannidis, 2005; Reis-Filho *et al*, 2006b).

Chromosomal comparative genomic hybridisation (cCGH) has been widely used to analyse the pattern of unbalanced genomic aberrations in breast cancer (Roylance *et al*, 1999; Buerger *et al*, 1999a, 1999b; Albertson, 2003; O’Connell, 2003; Reis-Filho *et al*, 2005a). More recent studies have employed higher resolution array-based CGH (aCGH) showing the enormous complexity of breast cancer genomes. Nonetheless, these studies have consistently reported the same regions of gain (1q, 8q, 11q, 17q, 20q) and loss (8p, 9p, 13q, 16q) (Rennstam *et al*, 2003; Loo *et al*, 2004; Naylor *et al*, 2005; Nessling *et al*, 2005). Unbalanced chromosomal aberrations and changes in gene copy numbers have been reported as a mechanism for acquired drug resistance to chemotherapy in cell line studies (Leyland-Jones *et al*, 1999; Yasui *et al*, 2004). However, no genome-wide analysis addressing response prediction by aCGH in breast cancer patients has been published.

The primary aim of this study was to determine whether aCGH could be used to identify unbalanced genomic changes predictive of response to preoperative doxorubicin and cyclophosphamide (AC) combination. We also sought to investigate the hypotheses that resistance to chemotherapy could be driven either by selection of chemotherapy resistant populations of neoplastic cells or by the induction of specific genomic aberrations.

## MATERIALS AND METHODS

### Patients and samples

Patients were recruited and treated at the Royal Marsden Hospital (Sutton and London, UK). Eligible patients were those undergoing neoadjuvant adriamycin and AC chemotherapy treatment at doses of 60 and 600 mg m^−2^, respectively, three weekly, for a clinically measurable breast cancer. Approval by the Royal Marsden Hospital Clinical Research and Ethics committees was granted and written consent was obtained in all cases. Patients were offered neoadjuvant treatment for one of several standard indications including locally advanced or inflammatory breast cancer, high tumour to breast size ratio and tumours located close to the nipple. The majority of the patients were from a previously published study on RNA expression profiling (Cleator *et al*, 2006).

Diagnosis was confirmed on core-cut biopsies subjected to routine histological analysis. Patients who demonstrated at least a partial clinical response received six cycles of treatment before local treatment. Patients with no or only marginal response after three or four cycles, proceeded directly to local treatment or were commenced on alternative systemic treatment (docetaxel).

Clinical size of tumour was recorded as the largest diameter and a diameter perpendicular to this. This was recorded before commencement and at completion of treatment. Clinical response was categorised as follows: no palpable abnormality after treatment, complete clinical response (cCR); >50% reduction in the product of the bidimensional measurements, partial response (PR); <50% reduction in the product of bidimensional measurements was recorded as no change (NC); residual ill-defined thickening after a good response, minimal residual disease (MRD) (Cleator *et al*, 2006). No patients in the study demonstrated progressive disease, an increase of more than 25% in the product of bidimensional measurements. Those cases in which there was no residual invasive carcinoma at surgery were classified as a complete pathological response (pCR). Good responders were defined as pCR, cCR, MRD; poor responders were defined as PR or NC. Eight patients undergoing a complete clinical and radiological (on ultrasound) response received radiation only as local treatment. Therefore some of the cCRs may represent undocumented pCRs.

Research 14-gauge core biopsies were collected before commencing treatment and again at 21 days after first treatment. Samples were snap frozen in liquid nitrogen. All samples were thereafter coded using a study number as an identifier. Frozen cores were embedded in optimum cutting temperature embedding compound and sectioned at −20°C in a cryostat. Sections (5 *μ*m thickness) were taken for hematoxylin and eosin staining to assess histological character superficially from the core as soon as ‘full-face’ was reached. The percentage of cells comprising invasive malignant disease was recorded. For patients in whom multiple biopsies were available, that with the highest invasive content was used for microarray analysis. Samples with less than 20% invasive cancer content were excluded from the study. Eight micrometer thick, nuclear fast red-stained representative frozen sections from 44 patients with primary breast cancer obtained before a neoadjuvant combination of AC were microdissected. In 22 patients consent for a second research core needle biopsy was granted and the procedure was technically feasible; in 17 of these, samples obtained 3 weeks after first course of chemotherapy (before cycle two) contained proportions of neoplastic cells suitable for microdissection (i.e. >20% of neoplastic cells). Twenty-one surgical formalin-fixed paraffin-embedded resection samples taken after 4–6 cycles of AC and with sufficient amounts of residual neoplastic cells were retrieved from the pathology files of the Royal Marsden Hospital.

### Fluorescent *in situ* hybridisation

Fluorescent *in situ* hybridisation (FISH) analysis was performed on representative 4-*μ*m sections using PathVysion HER-2 DNA probe mixture containing a HER-2 DNA probe (190 kb Spectrum Orange directly labelled DNA probe) and the CEP 17 DNA probe (5.4 kb Spectrum Green directly labelled fluorescent DNA probe specific for the chromosome 17 *α-*satellite DNA sequence) as described previously (Dowsett *et al*, 2003). A total of 60 cells were scored for red and green signals for each section and results were expressed as a ratio of red to green signals, with a ratio ⩾2.0 being considered amplified (Dowsett *et al*, 2003).

### Microdissection and DNA extraction

Frozen and formalin-fixed samples were subjected to aCGH analysis after microdissection of neoplastic cells with a sterile needle under a stereomicroscope (Olympus SZ61) from one to three consecutive 8 *μ*m nuclear fast red-stained sections (median=1). Estimated purity of tumour cells ranged from 75 to 100% (median=90%) (Supplementary Figure 1). DNA was extracted according to standard methods (Reis-Filho *et al*, 2005b). The DNA yield and purity was assessed by spectrophotometry and the DNA quality (DNA fragment size range) by agarose gel electrophoresis as described previously (Reis-Filho *et al*, 2005b).

### Microarray-based comparative genomic hybridisation

Microarray-based CGH was performed with in-house produced microarrays containing ∼5.8K bacterial artificial chromosome (BAC) clones, spaced out throughout the genome in ∼0.9 Mb intervals. The Breakthrough Breast Cancer Research Centre CGH microarray is composed of the same BAC clones as described by Reis-Filho *et al* (2005b).

Mapping of the BAC clones was retrieved from public sources and positioned according to the May 2004 build of the human genome sequence (hg17). When genomic positioning was dubious or conflicting, BAC end pair sequencing and FISH mapping was performed. Clones that either (i) showed poor quality end sequences or (ii) hybridised to multiple chromosomal locations or to a cytogenetic location inconsistent with their position in the sequence assembly were excluded from analysis. Bacterial artificial chromosome clones were spotted in triplicate onto Corning GAPSII-coated glass slides (Corning, NY, USA). Labelling of 250 ng of non-amplified DNA obtained from microdissected frozen sections or 1000 ng of DNA retrieved from microdissected formalin-fixed paraffin-embedded tissue sections, hybridisation and washes were carried out essentially as described previously (Reis-Filho *et al*, 2005b, 2006a; Natrajan *et al*, 2006).

Arrays were scanned with a GenePix 4000A scanner (Axon Instruments Inc., Union City, CA, USA); fluorescence data were processed with GenePix 4.1 image analysis software (Axon Instruments Inc.) as described previously (Reis-Filho *et al*, 2005b, 2006a; Natrajan *et al*, 2006).

### Data analysis

The log_2_ ratios were normalised for spatial and intensity-dependent biases using a two-dimensional Loess local regression. Experiment replicates (‘dye-swaps’) were collated, BAC clone replicate spots averaged, and clones excluded with poor reproducibility between replicates (s.d. >0.2). Samples with >70% missing/poor values were excluded, as were clones with missing/poor values in >70% samples. Missing data were imputed by k-nearest neighbours (Troyanskaya *et al*, 2001) and clones with no mapping information (May 2004 build of the human genome sequence, hg17) were excluded. A total of 5001 different BAC clones were used in the final analysis.

### Statistical analysis

All data transformation and statistical analysis were carried out in R 2.0.1 (http://www.r-project.org/), BioConductor 1.5 (http://www.bioconductor.org/), making extensive use of modified versions of the package aCGH in particular (Paris *et al*, 2004; Natrajan *et al*, 2006) and S-Plus (version 6.2.1). The log_2_ ratio of each BAC clone in each tumour sample was centred by the median of each case. Thresholds for determining copy number gains and losses were determined as exceeding ±3.0 × s.d. of the mean of these control experiments (log_2_ ratio of ±1). For identification of DNA copy number alterations, data were smoothed using a local polynomial adaptive weights smoothing procedure for regression problems with additive errors (Hupe *et al*, 2004; Natrajan *et al*, 2006).

Associations between genomic loci were assessed by calculating Pearson's correlations between thresholded values for each clone, assigned as 1, 0 or −1 for gain, NC, or loss in copy number. Thresholded data for each clone were also used for categorical analysis using a Fisher's exact test adjusted for multiple-testing with a correction for multiple testing using the step-down permutation procedure maxT, providing strong control of the family-wise type I error rate.

## RESULTS

### Patient, tumour and biopsy characteristics

A total of 44 patients were included in the study. Patient and tumour characteristics are summarised in Table 1 according to clinical response. Median tumour diameter was 4 cm (range 2–10). Two patients had inflammatory breast cancer (T4d). Median follow-up was 24 months (range, 9–38 months).

Of the 44 patients, 24 (55%) demonstrated a ‘good’ and 20 (45%) a ‘poor’ clinical response. Four patients (9%) were documented as undergoing a pathological Complete Response (pCR), 17 (37%) underwent a cCR and three (7%) had ill-defined thickening (MRD) at end of treatment; eight (19%) had a PR and 12 (28%) showed NC.

### Validation of aCGH method

DNA was extracted from 61 frozen samples. The median DNA yield obtained per sample was 660 ng (range 152–3188 ng). Owing to constraints in amount of tumour material available from frozen core biopsies after manual dissection, we assessed the reproducibility and quality of profiles obtained with 250, 500 and 1000 ng of tumour DNA. All DNA concentrations showed optimal results, with the Pearson's coefficient of correlation ranging from 0.93 to 0.96 *(*data not shown). Biological replicates of aCGH experiments also demonstrated high reproducibility: two independent research core biopsies were obtained before neoadjuvant chemotherapy for five patients. DNA extraction and CGH arrays were performed separately for these paired samples. For each of the matched pairs, the Pearson's correlation coefficient ranged from 0.64 to 0.86 (mean 0.78, data not shown). In six cases, *HER2* amplification was defined by aCGH. Fluorescent *in situ* hybridisation analysis confirmed the results in all cases, providing further evidence to support the validity of the aCGH analysis methods employed in this study (data not shown).

For six cases, high-resolution cCGH was performed and the genetic profiles compared with those obtained with aCGH. Correlation was good-to-excellent, with correlations for low-level gains and deletions >20 Mb and any amplification ranging from 60 to 87.5% (median=79.5%, mean=77.8%, data not shown). However, aCGH was more sensitive in detecting small losses and low-level gains than HR-CGH.

### Genomic alterations in 44 pre-chemotherapy breast cancer samples

**Figure 1:**
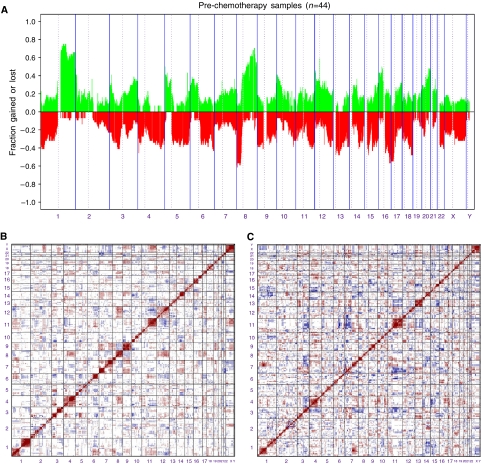
Frequency of copy number changes in 44 invasive breast carcinomas. (**A**) Overall frequency of DNA copy number alterations found in 44 invasive breast carcinomas as defined by aCGH. The proportion of tumours in which each clone is gained (green bars) or lost (red bars) is plotted (*y* axis) for each BAC clone according to genomic location (*x* axis). Vertical dotted lines represent chromosome centromeres. (**B**) – Pearson's correlation matrix of all thresholded aCGH data for 29 ER-positive, invasive breast carcinomas. Strong positive correlations are plotted as dark red, strong negative correlations as dark blue. Note the associations between chromosomes 1p/8p, 1p/12q, 4q/8p, 7p/22, 8q/12q and 11q/12q. Bacterial artificial chromosome clones are plotted in genome order. (**C**) Pearson's correlation matrix of all thresholded aCGH data for 15 ER-negative, invasive breast carcinomas. Strong direct plotted as dark red, strong negative correlations as dark blue. Note the associations between chromosomes 1p and 7q, 16p and 17q, and strong inverse correlations between 1q and 14q, 2q and 9q, 4q and 15q, 6q and 11q, 7p and 11q, 7p and 12q, 9p and 16p, and 13q and 15q. Bacterial artificial chromosome clones are plotted in genome order.

To identify genomic regions harbouring recurrent unbalanced genomic changes, we plotted the frequency of tumours showing gain or loss for each BAC across the genome (Figure 1A). The most frequent (>30%) genomic changes (Table 2) comprised gains of 1q (66%), 5p (32%), 8q (70%), 16p (36%) and 20q (41%) with the smallest regions of genomic gain on 1q31.1–1q31.2 and 1q22–1q25.3, 5p15.31–5p15.33, 8q23.1–8q25.1, 16p11.2–16p12.2 and 20q13.13–20q13.33, respectively. Losses were observed on 4q (39%), 8p (50%), 9p (36%), 11q (32%), 13q (36%), 16q (52%) 17p (50%) and 18q (39%) with the smallest regions of deletion on 4q32.3–4q33, 8p21.1–p23.3, 9p22.2–9p24.3, 11q23.1–11q25, 13q14.11–13q14.3, 16q23.1–q24.3, 17p12–17p 13.3 and 18q22.1–q23. In addition to the large regional alterations, the resolution of the BAC array allowed us to map smaller regions of gain or loss. Bacterial artificial chromosome clones gained or deleted in >30% of the tumours are described in Supplementary Tables 1 and 2, respectively.

### Comparison of genomic alterations in different phenotypes of breast cancer

On the basis of three distinguishing phenotypic characteristics (ER, HER2 and histological type), we characterised genetic alterations that might be associated with subtypes of breast cancer on the 44 pretreatment biopsies. Oestrogen receptor (ER)-negative tumours (*N*=15) were characterised by significantly more chromosomal changes than ER-positive tumours (Table 3 and Supplementary Figure 2). Subgroup analysis showed that the following chromosomal changes were more frequently associated with ER negativity: gains on 1p, 2p, 2q, 6q, 7q, 9p, 11q and 19q with large regions on 1p31–1p34.2, 7q32–7q36 and losses on 4p, 4q, 5p, 5q, 12q and 17q as well as large regions such as the whole long arm of chromosome 5 and whole chromosome 4.

Pearson's correlation analysis of the subset of ER-positive tumours (*n*=29) revealed chromosomal alterations that coexist in this group of tumours. The heat map in Figure 1B shows regions of positive (change in same direction: red) and negative (change in opposite direction: blue) correlations. Relatively large regions of correlation were seen between changes in copy number of BACs on chromosome 1p and 8p (*P*<0.01), 3p and 4p (*P*<0.001), 4q and 8q (*P*<0.01) and 8q and 12q (*P*<0.01) were observed, as were strong inverse correlations between 1p and 12q (*P*<0.0005), 7p and 22 (*P*<0.001) and 11q and 12q (*P*<0.001) (Figure 1B). Pearson's correlation matrix of ER-negative tumours revealed strong direct associations between 1p and 7q (*P*<0.001), 16p and 17q (*P*<0.001), and strong inverse correlations between 1q and 14q (*P*<0.005), 2q and 9q (*P*<0.001), 4q and 15q (*P*<0.001), 6q and 11q (*P*<0.001), 7p and 11q (*P*<0.001), 7p and 12q (*P*<0.01), 9p and 16p (*P*<0.001) and 13q and 15q (*P*<0.005) (Figure 1C).

Comparing invasive lobular carcinomas (*n*=7) with non-lobular cases (37 cases), 16q loss (from 16q12.1 to 16q24.3) was significantly associated with lobular phenotype (unadjusted Fisher's exact test <0.01) (Supplementary Table 3 and Supplementary Figure 3). This region contains a number of cadherin genes (CDH1, CDH3, CDH5, CDH 8, CDH 11). Gain of 8q21.11–q24.23 was significantly associated with the ductal phenotype (Supplementary Table 3 and Supplementary Figure 3).

Comparison between the six cases with HER2 amplification on 17q11.2 with the 38 HER2-negative cases revealed more frequent gains of 6p25.2–p24.2, 17q12 and Xp22.33–p11.36, and loss of 18p11.31 (Supplementary Table 3 and Supplementary Figure 4). On the other hand, loss of 16q23.3–q24.1 was more frequently observed in HER2 – tumours. This would be expected given that all HER2+cases were of histological grade III and loss of 16q is significantly less frequently found in this group of tumours (Reis-Filho *et al*, 2005a; Simpson *et al*, 2005).

### Prediction of tumour response

**Figure 2:**
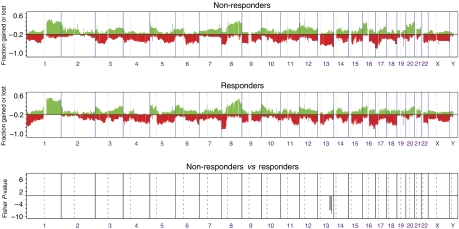
Categorical analysis of copy number gains and losses between non-responders (*N*=20) and responders (*N*=24). Fisher's exact tests are carried out on the segmented values for each clone, and those with a *P*-value of less than 0.01 are plotted (inverse log10, *y* axis) according to genomic location (*x* axis).

Comparison of the molecular genetic profiles of objective clinical responders (complete and partial clinical responders, *N*=24) with those of non-responders revealed loss of a 13.3 Mb region on 13q31.1–13q32.2 to be the only significant difference (Figure 2). This region includes the genes *SLITRK6*, *SLITRK5*, *GPC5*, *GPC6*, *DCT*, *TGDS*, *SOX21*, *ABCC4*, *CLDN10*, *DZIP1*, *DNAJC3*, *UGCGL2*, *HS6ST3*, *HSP90AB6P*, *OXGR1*, *MBNL2* and *RAP2A*. In addition, this region also encompasses the micro RNA cluster miR-17-92, which is reported to induce tumour cell growth and to be overexpressed and sometimes amplified in aggressive forms of lung cancer (Hayashita *et al*, 2005).

### Comparison of pre-chemotherapy samples to D21 samples, before cycle two of AC

**Figure 3:**
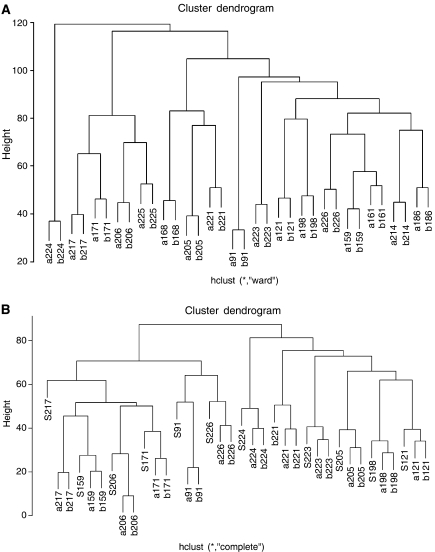
Hierarchical clustering analysis (Ward's method/Euclidean distance) of matched pre- and post-chemotherapy samples (17 patients) (**A**) and pre-chemotherapy, post-chemotherapy and surgical samples (12 patients) (**B**). (b – before chemotherapy; a – after chemotherapy (day 21); s – surgical biopsy).

Unsupervised hierarchical clustering analysis based upon genetic alterations on all chromosomes showed that all pre- and D21 post-biopsies clustered together (*N*=17 cases) (Figure 3A). There were no significant differences in the profiles between poor (10 cases) and good responders (seven cases) (*P*=0.95, Kolmogorov–Smirnov test). The comparison of the pattern and frequency of unbalanced genomic changes detected by aCGH analysis in matched pretreatment and D21 biopsies revealed no significant differences (multi-Fisher's exact test, data not shown).

### Comparison of pre-chemotherapy samples to surgical samples, after 4–6 cycles of AC

Twenty-one surgical samples out of 44 cases were available (48%). All but three segregated with the respective pre-chemotherapy sample by unsupervised hierarchical clustering (Figure 3B). For 12 cases, pre-, D21 and at surgery samples were available. Unsupervised hierarchical clustering analysis based upon genetic alterations on all chromosomes was performed. All 12 ‘triplicates’ clustered together.

**Figure 4:**
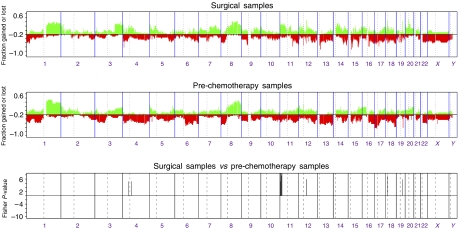
Categorical analysis of copy number gains and losses between matched pre-chemotherapy and surgical tumour samples (21 patients). Fisher's exact tests are carried out on the segmented values for each clone, and those with a *P*-value of less than 0.01 are plotted (inverse log10, *y* axis) according to genomic location (*x* axis).

Comparison between the genomic profiles obtained with DNA extracted from matched frozen and formalin-fixed pretreatment core biopsies were performed (*n*=2). The profiles showed a good concordance (*r*^2^>0.8). Subsequently, a comparison of the aCGH profiles of 21-matched pretreatment core biopsies and the respective excision specimens obtained after 4 months of neoadjuvant chemotherapy revealed the presence of gains of 4q13.1 (9/21, 43%), 11p15.2–p15.5 (14/21, 66%), 12q13.3 (14/21, 66%), 18p11.21 (12/21, 52%) and 19q13.2 (12/21 52%) in the excision specimens (Figure 4 and Supplementary Table 4). After FDR adjustment, only the gain on 11p15.2–p15.5 retained its statistical significance. Although we cannot completely rule out that this change could result from the comparison between DNA extracted from frozen and formalin-fixed samples, this 14.3 Mb telomeric region encompasses interesting oncogene candidates, such as *H-RAS*, a bona fide oncogene, and *IGF2*, which has been reported to show loss of imprinting in up to 30–60% of breast cancers (McCann *et al*, 1996; Wu *et al*, 1997). In the pre-chemotherapy samples, gain on 11p15.2–p15.5 was observed in five out of 10 good responders and in two out of 11 poor responders (not significant, Fisher's exact test).

## DISCUSSION

In the present study, the frequency and pattern of unbalanced genomic aberrations were similar to those described in previous studies where genome-wide changes were analysed by means of chromosomal CGH (Tirkkonen *et al*, 1998; Buerger *et al*, 1999a, 1999b; Roylance *et al*, 1999; Seute *et al*, 2001; Rennstam *et al*, 2003) and aCGH (Callagy *et al*, 2005; Naylor *et al*, 2005; Nessling *et al*, 2005). Recurrent gains on chromosome 1q, 8q, 11q, 17q and 20q and losses on 6q, 8p, 9p, 13q and 16q were the most prevalent changes. In addition, we confirmed the association between ER positivity and gain of 1q coupled with loss of 16q (Farabegoli *et al*, 2004; Reis-Filho *et al*, 2005a; Simpson *et al*, 2005) and the more prevalent deletions of 4p16 and 4p15, 5q and 17p11.2 in ER-negative tumours (Loo *et al*, 2004). In contrast to previous studies (Loo *et al*, 2004), gains of 8q24.1 (*MYC*) and 17q12 (*HER2*) were not significantly more frequent in ER-positive tumours. This is expected, given that only breast carcinomas of histological grades 2 and 3 were present in the population and gains of 8q are rather frequent in grade 2 and 3 breast cancers (Buerger *et al*, 1999a, 1999b; Roylance *et al*, 1999).

The comparison between the genomic profiles obtained for ductal and lobular carcinomas were also in agreement with previous studies (Buerger *et al*, 1999b; Shelley Hwang *et al*, 2004; Reis-Filho *et al*, 2005a; Simpson *et al*, 2005; Stange *et al*, 2006): gain of 1q and deletions of 16q were the most prevalent changes in lobular carcinomas, whereas gain of 8q was significantly more frequent in grade 2 and 3 ductal carcinomas. However, we could define the smallest region of overlap of the deletions of 16q, which mapped to 16q21–q22.1 and encompassed the region of the cadherin gene cluster, and the gain of 8q, which encompassed two regions 8q13.2–q21.13 and 8q21.3–qtel (Supplementary Table 3). The most significant gain comparing ductal carcinoma with lobular carcinomas mapped to 8q24.11 (117.8–118.0 Mb), which encompasses *RAD21* and eucaryotic translation initiation factor 3, subunit 3 gamma (*EIF3S3*). *RAD21* is believed to function in sister chromatid alignment as part of the cohesin complex and also in double-strand break repair and influences cellular proliferation (Atienza *et al*, 2005), whereas *EIF3S3* is reported to be amplified and overexpressed in up to 20% of breast carcinomas (Nupponen *et al*, 1999). Our observations are consistent with previous studies on breast cancer, confirming the robustness of our aCGH protocol, the validity of our analysis method and the likelihood that we have a representative set of tumours.

Several attempts have been made to predict clinical or pathological response to neoadjuvant chemotherapy in breast cancer using gene expression arrays. Gene ‘signatures’ or predictors have been devised for several chemotherapy regimens, including paclitaxel followed by fluorouracil, AC, AC/doxorubicin-docetaxel and taxane only chemotherapy (Chang *et al*, 2003, 2005b; Ayers *et al*, 2004; Hannemann *et al*, 2005; Cleator *et al*, 2006). Although these results are promising, the exceedingly small sample size and limitations with the current technology and analysis methods have so far precluded definitive conclusions (Brenton *et al*, 2005; Reis-Filho *et al*, 2006b).

We have recently reported a similar expression profiling study (Cleator *et al*, 2006) that included the 44 patients studied here plus a small number of others for whom pretreatment tissue for DNA analysis was not available. Neither unsupervised nor supervised methods could separate the responders from non-responders. In the current study, using the same cohort of patients, which included 24 good clinical responders, aCGH analysis revealed a deletion of a large region from 13q31.1 to 13q34 as the only significant copy number change associated with response to chemotherapy. Although this statistical association lost its significance after correction for multiple comparisons, the correction method, which we adopted may be too conservative, given that unbalanced chromosomal aberrations usually encompass more than one BAC clone. One of the genes deleted in this region is a subtype of heat-shock protein 90, *HSP90AB6*. HSP 90 is a molecular chaperone whose association is required for stability and function of multiple signalling proteins that promote cancer cell growth and/or survival (Chen *et al*, 2005). Further studies with a larger sample size focusing on this particular region are warranted. We cannot exclude the possibility that analysis similar to ours of a larger series of samples might identify other DNA aberrations of importance for response to AC chemotherapy. It is however likely that if these were present in a large proportion of the population they would have been uncovered by the present analysis.

Sequential sampling of tumour during neoadjuvant treatment can be used to detect gene expression modification induced by therapy as we have demonstrated with endocrine therapy (Mackay *et al*, 2005). This was demonstrated for chemotherapy by Hannemann *et al* (2005), who observed that tumours that responded to neoadjuvant chemotherapy showed dramatic changes in their expression profiles when compared to the changes observed in non-responders (Hannemann *et al*, 2005). On the other hand, a comparison between the transcriptomic profiles of tumours subjected to taxane-based neoadjuvant chemotherapy before and 3 months after treatment revealed strikingly different patterns, independent of initial sensitivity or resistance (Chang *et al*, 2005b).

Cell line studies have demonstrated that changes in gene copy numbers may lead to acquired resistance to chemotherapy (Leyland-Jones *et al*, 1999; Shimizu *et al*, 2002; Yasui *et al*, 2004). Specific gains of genetic material mapping to multi-drug resistance (MDR) gene *MDR1* locus have been reported in drug resistant cell lines (Shimizu *et al*, 2002; Kuwano *et al*, 2003). Recurring amplicon 7q11.2–q21 identified by CGH in doxorubicin-resistant hepatocellular carcinoma cell lines coincided with the localisation of *MDR1* (Pang *et al*, 2005). Induction of DNA damage response genes such as p21 were obtained by doxorubicin in breast cancer cell lines (Troester *et al*, 2004). Both chromosome 7 alterations and several cytogenetic changes involving the 7q21 locus are associated with the development of MDR in sarcoma cells (Chen *et al*, 2002). Analysis of genomic amplifications and deletions revealed specific genetic alterations common to both intrinsic and acquired doxorubicin resistance including *ABCB1*, *PGY3* (*ABCB4*) and *BAK* (Turton *et al*, 2001).

Most patients show some tumour shrinkage with neoadjuvant chemotherapy and for many there may be profound loss of malignant cells with some showing a pCR. Characterisation of the residual cells may be expected to a better understanding of the causes of resistance and allow the identification of the means to overcome the resistance. Given the molecular heterogeneity between breast carcinomas and the composition of most breast cancers being of multiple non-modal clones, we hypothesised that the cell loss might lead to selection of breast cancer cell lineages that were resistant to chemotherapy by virtue of specific DNA alterations. Few studies have previously addressed this issue. A very small study, involving CGH of just four tumours from breast cancer patients after neoadjuvant chemotherapy (Fazeny-Dorner *et al*, 2003) showed typical DNA imbalances for ductal breast cancer. Three patients showed involvement of several regions bearing genes of drug resistance (*MDR1*, *BCRP*, *MRP1*, *RFC1*); the fourth patient displayed an amplification in the region of MYC. In our study, the hierarchical clustering of sequential samples at baseline, 3 weeks and at surgery indicated that any changes induced in DNA profiles by therapy were modest compared with the differences that were present between the patients. This interpretation was supported by the observation that statistically significant chemotherapy-driven genomic changes were not detected within 3 weeks (i.e. after a single course of chemotherapy). Comparison of the pretreatment and the tumour specimens excised after 4 months of neoadjuvant chemotherapy revealed the appearance of genomic gains on 11p15.2–p15.5. Given the experimental design of our study, we cannot define whether these amplifications were chemotherapy induced or were present in a non-modal population of the primary tumour and selected by chemotherapy, as the majority of surgical samples by definition were obtained from poor responders (66%). Interestingly, pathological relaxation of the imprinting pattern in this region is reported to be found in 30–60% of breast cancers (McCann *et al*, 1996; Wu *et al*, 1997). However we did not find a correlation between amplification on 11p15.5 and clinical response (Han *et al*, 2006). Furthermore, these changes on surgical samples, need to be cautiously interpreted as surgical samples were subjected to different fixation conditions (Devries *et al*, 2005). Thus in contrast to observations with cancer cell lines, we did not find large or frequent chemotherapy-induced acquired genomic changes. Given that aCGH provides an average of the pattern of genomic gains and losses in the cell population studied, chemotherapy-driven losses or low-level gains occurring in non-modal clones are unlikely to be detected.

In conclusion, array CGH is a powerful method for the genome-wide detection of chromosomal imbalances and allowed us to detect molecular genetic aberrations associated with specific breast cancer subgroups (ductal *vs* lobular, ER-negative *vs* ER-positive tumours). A molecular genetic profile specific of good responders to neoadjuvant chemotherapy was not detectable in our series. Chemotherapy-driven genomic changes were not detected following 3 weeks of treatment and only a single change after completion of treatment. The hypothesis of resistance to neoadjuvant chemotherapy by the selection of non-modal cell lineages, which differ by gene amplifications or losses is not supported by our results.

## Figures and Tables

**Table 1:** Patient and tumour characteristics

**Patients**	**Responders (*n*=24)**	**Non-responders (*n*=20)**	***P*-value**
*Patient age*			
⩽40	5	2	0.57^*^
>40	19	18	
			
*Menopausal status*			
Post	12	6	0.22^*^
Pre	12	13	
		1 unavailable	
			
*Tumour size*			
T2	15	10	0.40^*^
T3/T4	9	10	
			
*Nodal status*			
N0	15	14	0.60^*^
N1/N2/N3	9	6	
			
*HER2 – IHC*			
Negative	17	19	0.03^*^
Positive	7	1	
			
*HER2 – FISH*			
Negative	19	18	0.28^*^
Positive	5	1	
		1 unavailable	
			
*Lympho-vascular invasion*			
Absent	19	10	0.04^*^
Present	5	10	
			
*Histological type*			
Invasive ductal	19	18	0.39^**^
Lobular	5	2	
Medullary	1	0	
Metaplastic	0	1	
			
*Histological grade*			
I	0	0	
II	8	10	0.26^**^
III	16	10	
			
*Oestrogen receptor*			
Negative	8	7	0.6^*^
Positive	16	13	

FISH=fluorescent *in situ* hybridisation.

^*^: Fisher's exact test; ^**^: *χ*^2^ test.

**Table 2:** Recurrent gains and losses of genomic material in >30% of the samples

**Gain region**	**Start (Mb)**	**End (Mb)**	**Loss region**	**Start (Mb)**	**End (Mb)**
1p12–qtel	119.8	245.2	1p31.1	75.3	78.3
2p25.3–p25.1	0.1	8.9	1p31.1–p21.3	79.2	97.8
3q26.1–q29	163.8	198.3	1p32.1–p31.1	58.7	69.1
5p13.3–p12	31.9	44.9	1p36.33–p34.2	1	42.9
5p15.33–p14.3	0.4	20.6	3p24.3–p13	20.2	74.2
6p12.3–p12.1	49.6	53.5	4p15.31–p15.2	18	26.6
6p24.3–p22.1	7.3	31.2	4p16.1–p15.33	7.2	15.4
7q34–q35	142.2	144.8	4q21.23–q35.2	85.1	190.9
8p12	36.8	37.9	5q11.2–q13.2	54.7	71
8p12–qtel	38.3	144.9	5q13.2–q35.1	72.4	170.2
10p15.3	0.1	1.2	6q13–q16.2	74.5	99.7
10p15.3–p12.1	2.2	27.8	6q21–23.2	105.8	131.9
12p11.22–q11	29.5	36.9	6q25.1–qtel	151.7	170.4
12p12.1–p11.22	21.3	29.3	8p23.3–p12	0.3	36.7
12p13.2–p12.1	9.8	21.3	9p24.3–q21.1	0.1	68.5
12p13.33–p13.31	0.1	8.1	9q22.2–q31.1	87.4	99.8
12q13.3–q14.1	56.5	58.3	10p15.3	0.9	1.2
12q15–q21.1	67.6	71.2	10q25.3–qtel	118.4	135.1
12q24.3–q25.1	67.1	71.8	11p15.2	14.8	15.2
12q25.1–qtel	73	78.2	11q14.2–q14.3	86.4	88.9
15q26.2–q26.3	96.3	97.7	11q14.3–qtel	91.9	133.7
16p13.3–p11.2	0.9	32.9	12q22–q24.23	94.9	118.2
20p11.21	23.3	26.2	12q24.31–qtel	121.6	132.2
20q11.21–q13.33	30	62.4	13q12.11–q31.1	18.3	85.2
			13q33.1–qtel	100.2	114.1
			14q23.3–q24.3	63.1	76.9
			14q31.1–q32.11	79.5	87.9
			14q32.12	90.8	91.4
			15q11.2–q14	20.5	33.3
			15q14	33.4	37.5
			15q21.3–q24.3	55.1	75.5
			15q25.1	78.6	79.3
			15q25.2–q26.1	81.6	89.6
			15q26.1–q26.2	91.4	94.6
			15q26.3–qtel	97.9	99.9
			16q12.1–qtel	46.4	88.5
			17p13.3–q11.2	1.1	34.1
			18p11.31–p11.23	6.1	7.6
			18q11.2–q12.1	20.6	25.6
			18q12.3–qtel	40.6	75
			19q13.31–q13.32	48.9	50.1
			22q11.21–q13.33	17.3	49.2
			23q22.1–q28	98.6	146.7

**Table 3:** Copy number changes significantly more prevalent in ER-negative compared to ER-positive tumours

**ER negative vs ER positive**	**Start**	**End**	**Genes (regions <15 Mb)**
*Gain*			
1p21.1–p13.1	102.8	117.2	
1p31.3–p21.3	63.2	97.8	
1p34.3–p32.3	39	55.1	
2p11.2–q11.2	85.6	97.9	
2p16.1–p14	58.3	65.4	*BCL11A, PAPOLG, REL, PEX13, AHSA2, USP34, XPO1, CCT4, COMMD1, B3GNT1, TMEM17, EHBP1, OTX1, MDH1, UGP2, VPS54, PELI1, AFTIN, SERTAD2, SLC1A4, CEP68, RAB1A, ACTR2, SPRED2*
2p25.3–p25.1	0.4	12	
2q24.1	158	159.7	*ACVR1C, ACVR1, UPP2, PKP4, TANC1*
6q23.2	131.3	134.9	*AKAP7, ARG1, CRSP3, ENPP3, OR2A4, CTAGE4, ENPP1, CTGF, MOXD1, STX7, TAAR9, TAAR8, TAAR6, TAAR5, TAAR2, TAAR1, VNN1, VNN3, VNN2, RPS12, EYA4, TCF21, TBPL1, SLC2A12, SGK*
7q32.3–q33	130.9	134	*PLXNA4B, CHCHD3, EXOC4, SLC35B4, AKR1B1, AKR1B10, BPGM*
7q34–qtel	141.3	158	
8q11.21	48.9	51.4	*MCM4, UBE2V2, EFCAB1, SNAI2*
9p24.1–p22.2	5.1	18	
11q24.1-qtel	121	133.7	
12q13.13	49.8	52.8	*POU6F1, DAZAP2, BIN2, ELA1, GALNT6, SLC4A8, SCN8A, ACVR1B, ANKRD33, ACVRL1, GRASP, NR4A1, KRT7, KRTHB1, KRTHB6, KRTHB3, KRTHB5, KRTHB4, KRTHB2, KRT6B, KRT6E, KRT6A, KRT5, KRT2A, KRT1, KRT1B, K22O, KRT3, KRT4, KRT8, KRT18, EIF4B, TENC1, SPRYD3, IGFBP6, SOAT2, CSAD, ZNF740, ITGB7, RARG, MFSD5, ESPL1, PFDN5, MYG1, AAAS, SP7, SP1, AMHR2, PCBP2, MAP3K12, TARBP2, NPFF, ATF7, ATP5G2, CALCOCO1, HOXC13, HOXC12, HOXC11, HOXC10, HOXC9, HOXC8, HOXC6, HOXC5, HOXC4*
19p13.11	19.2	21.3	*TM6SF2, GATAD2A, SF4, TSSK6, NDUFA13, CILP2, PBX4, EDG4, GMIP, ATP13A1, ZNF101, ZNF14, ZNF506, ZNF56, ZNF93, ZNF682, ZNF90, ENH5, ZNF486, ENT1, ZNF626, ZNF85, ZNF430, ZNF714, ZNF431, ZNF708*
19q13.2	44.1	44.9	*MRPS12, FBXO17, FBXO27, PAK4, IL28B, IL28A, IL29, LRFN1, GMFG, SAMD4B, PAF1, IXL, ZFP36, PLEKHG2, RPS16, SUPT5H, TIMM50, DLL3, SELV, LGALS13, LGALS14*
19q13.2–q13.31	45.2	48.8	*MAP3K10, TTC9B, AKT2, PLD3, HIPK4, PRX, SERTAD1, SERTAD3, BLVRB, SPTBN4, SHKBP1, LTBP4, NUMBL, ADCK4, ITPKC, SNRPA, RAB4B, EGLN2, CYP2A7, CYP2B, CYP2B6, CYP2G1P, CYP2A13, CYP2F1, CYP2S1, AXL, HNRPUL1, TGFB1, BCKDHA, EXOSC5, CEACAM21, CEACAM4, CEACAM7, CEACAM5, CEACAM6, CEACAM3, LYPD4, DMRTC2, RPS19, CD79A, ARHGEF1, RABAC1, ATP1A3, GRIK5, ZNF574, POU2F2, DEDD2, ZNF526, GSK3A, ERF, CIC, PAFAH1B3, EGFL4, CNFN, LIPE, CEACAM1, CEACAM8, PSG3, PSG8, PSG1, PSG6, PSG4, PSG11, PSG5, PSG9, TEX101, LYPD3, PHLDB3, ETHE1, ZNF575, XRCC1, IRGQ, ZNF576*
			
*Loss*			
4p15.32–p15.2	16.9	26.6	*QDPR, LAP3, MED28, CND3, SLIT2, KCNIP4, GPR125, GBA3, PPARGC1A, DHX15, SOD3, LGI2, PI4K2B, ZCCHC4, ANAPC4, SLC34A2, Q9BRT5, RBPSUH, CCKAR, TBC1D19, STIM2*
4p15.33–p15.32	8.8	15.4	*DB131, GAK19, ENK19, DRD5, SLC2A9, WDR1, HS3ST1, HSP90AB2P, RAB28, BAPX1, FAM44A, Q6PID2, CPEB2, C1QTNF7, FBXL5, BST1, CD38*
4p16.3–16.1	3.5	8.6	
4q24–q34.3	106.7	178.4	
4q35.1–q35.2	186.4	188.8	*LRP2BP, ANKRD37, PDLIM3, SORBS2, TLR3, CYP4V2, KLKB1, F11, MTNR1A, FAT*
5p15.33	2.8	5	*IRX1*
5q11.1–q13.2	50.1	71	
5q13.2–q14.3	72.4	85.5	*BTF3, ANKRA2, UTP15, ENC1, HEXB, GFM2, TIP1, GCNT4, ANKRD31, HMGCR, COL4A3BP, POLK, SV2C, IQGAP2, F2RL2, F2R, F2RL1, S100Z, CRHBP, AGGF1, ZBED3, PDE8B, WDR41, OTP, TBCA, AP3B1, SCAMP1, LHFPL2, ARSB, DMGDH, BHMT2, BHMT, HOMER1, PAPD4, CMYA5, THBS4, SERINC5, ZFYVE16, MSH3, RASGRF2, CKMT2, ZCCHC9, ACOT12, SSBP2, ATG10, RPS23, XRCC4, CSPG2, HAPLN1, EDIL3*
5q15	92.7	96.1	*NR2F1, ANKRD32, MCTP1, FAM81B, ARSK, GPR150, SPATA9, RHOBTB3, GLRX, ELL2, PCSK1, CAST*
5q22.1–q23.3	110.9	131.6	
5q23.3–q33.1	132.2	148.5	
5q33.1–q35.2	150.2	175.5	
12q13.11	44.7	44.9	*SLC38A1*
12q13.12–q13.13	46.3	50.6	*PP11, RAPGEF3, HDAC7A, VDR, TMEM106C, COL2A1, SENP1, PFKM, ASB8, OR10AD1, H1FNT, ZNF641, ANP32D, OR8S1, LALBA, CCNT1, ADCY6, CACNB3, DDX23, RND1, CCDC65, FKBP11, ARF3, WNT10B, WNT1, DDN, PRKAG1, MLL2, RHEBL1, DHH, LMBR1L, TBAK, TBA3, TUBA6, PRPH, TROAP, C1QL4, SPATS2, KCNH3, MCRS1, PRPF40B, FMNL3, TEGT, FAIM2, AQP2, AQP5, AQP6, RACGAP1, SMARCD1, GPD1, LASS5, LIMA1, LARP4, DIP2B, ATF1, TMPRSS12, METTL7A, SLC11A2, LETMD1, TAI12, TFCP2, POU6F1, DAZAP2, BIN2, ELA1, GALNT6, SLC4A8, SCN8A, ACVR1B, ANKRD33, ACVRL1*
13q14.2	45.7	47.8	*LRCH1, ESD, HTR2A, SUCLA2, NUDT15, MED4, ITM2B, RB1*
13q21.1–q21.31	57.1	60.8	*PCDH17, DIAPH3, TDRD3*
17p11.2–q11.2	21.2	30.3	*KCNJ12, FAM27L*
