# Supplementary figures and images for: Microarray-based comparative genomic hybridisation of breast cancer patients receiving neoadjuvant chemotherapy
Source: Br J Cancer. 2006 Nov 28;96(2):341–51. doi: 10.1038/sj.bjc.6603483 (PMC2359992; doi:10.1038/sj.bjc.6603483)

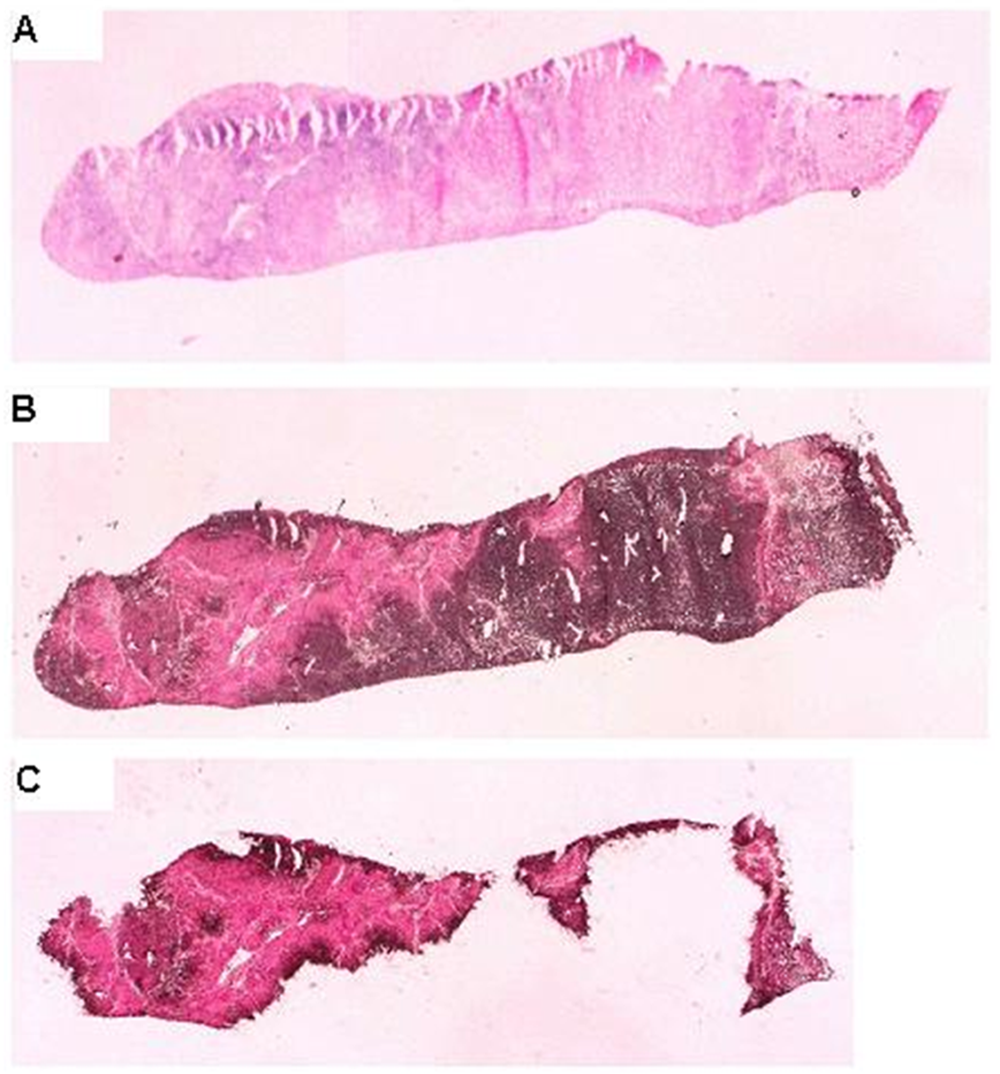

Supplement: Supplementary Figure 1 [file 6603483x1.tif]

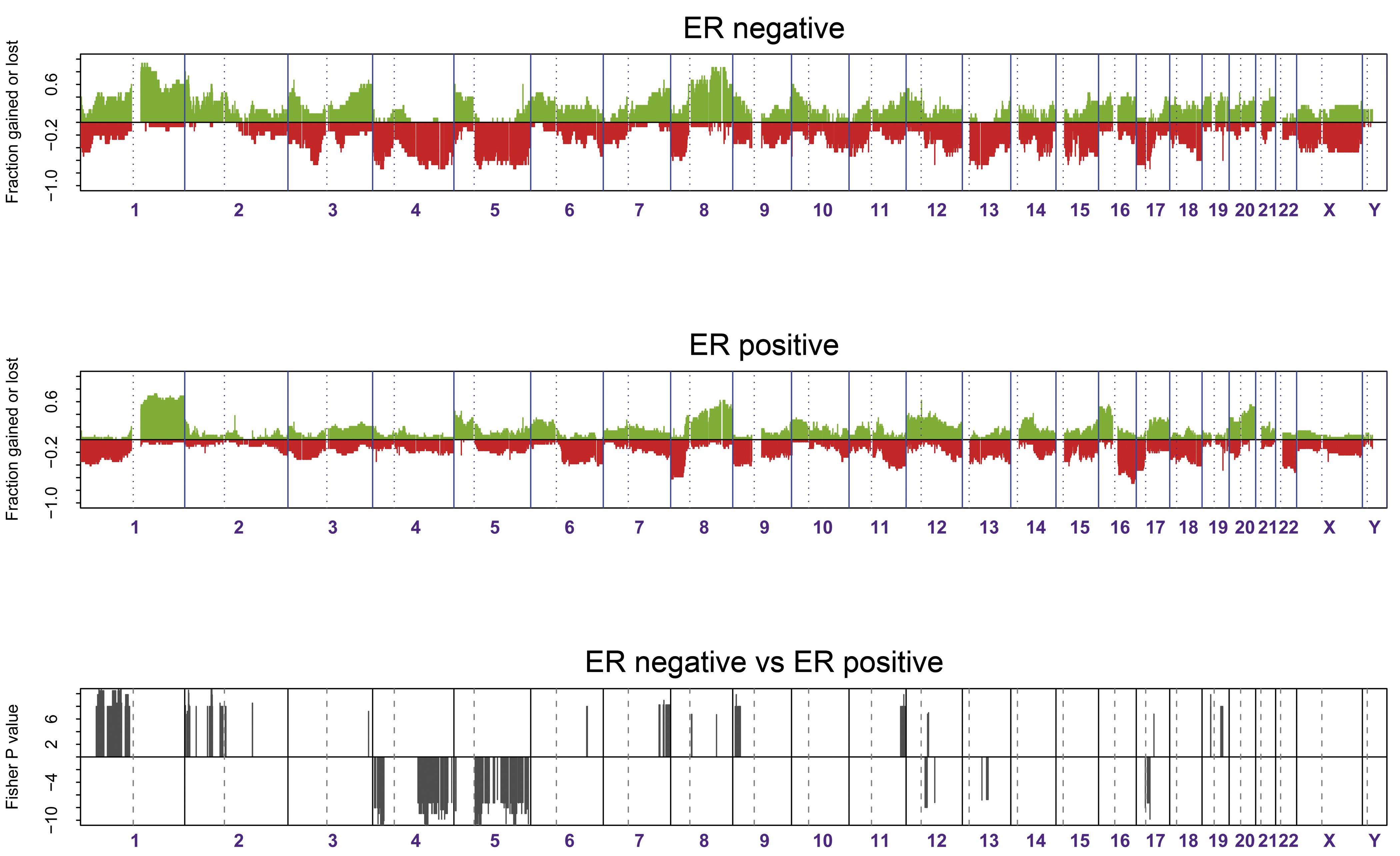

Supplement: Supplementary Figure 2 [file 6603483x2.tif]

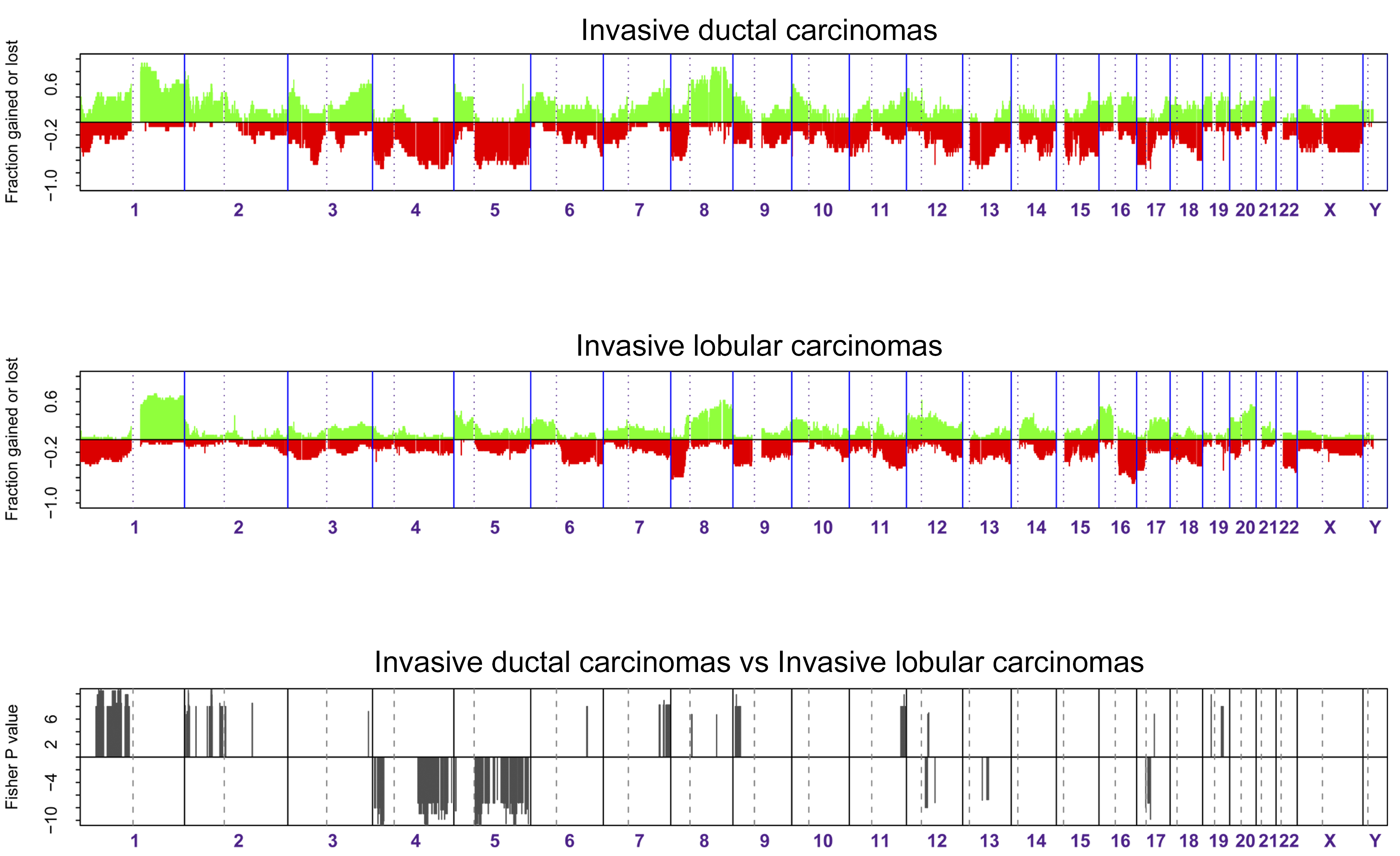

Supplement: Supplementary Figure 3 [file 6603483x3.tif]

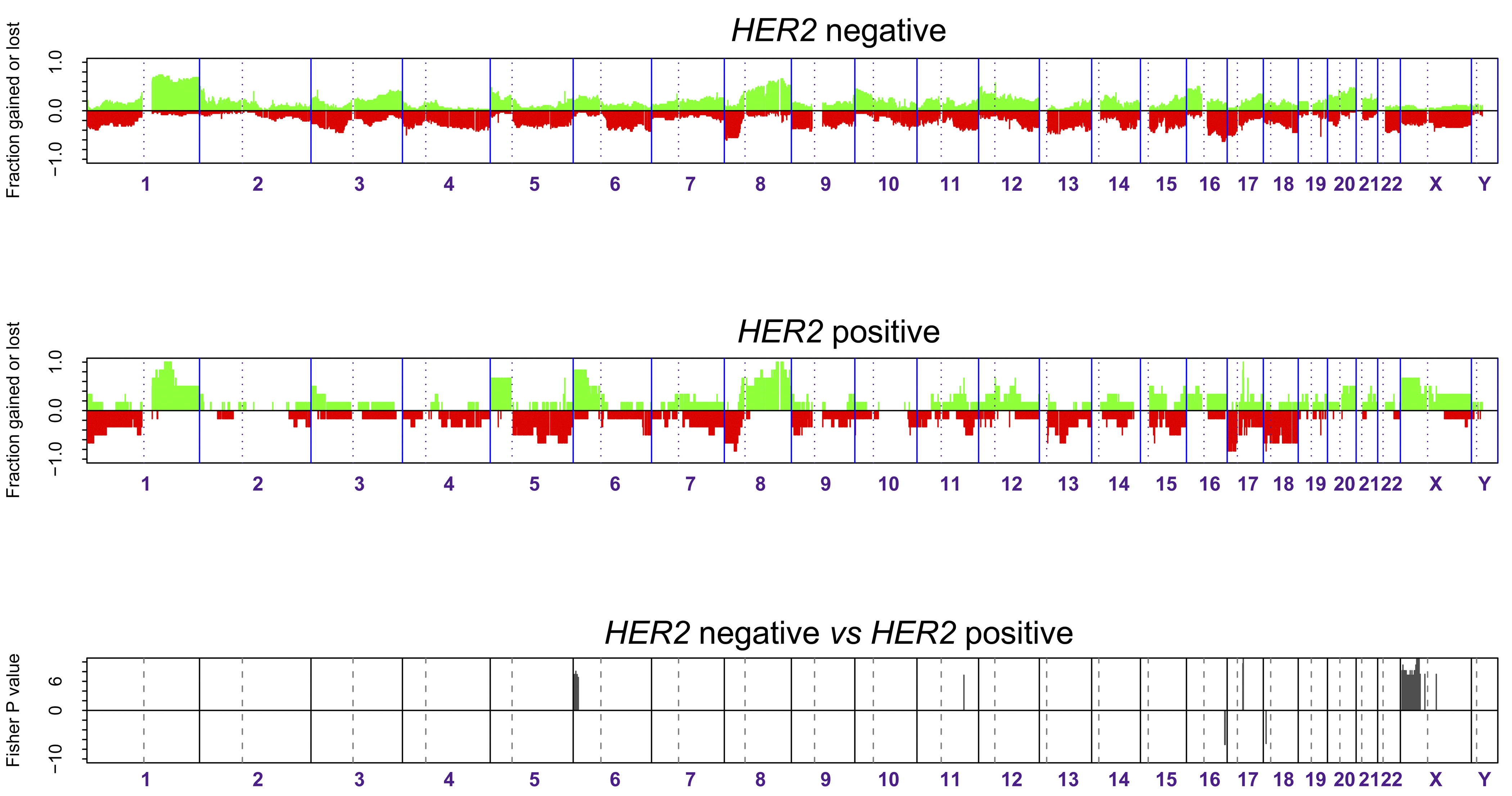

Supplement: Supplementary Figure 4 [file 6603483x4.tif]
